# Engaging stakeholders in the adaptation of the *Connect for Health* pediatric weight management program for national implementation

**DOI:** 10.1186/s43058-020-00047-z

**Published:** 2020-06-17

**Authors:** Meg Simione, Holly M. Frost, Rachel Cournoyer, Fernanda Neri Mini, Jackie Cassidy, Cassie Craddock, Jennifer Moreland, Jessica Wallace, Joshua Metlay, Caroline J. Kistin, Kerry Sease, Simon J. Hambidge, Elsie M. Taveras

**Affiliations:** 1 Division of General Academic Pediatrics, MassGeneral Hospital for Children, 125 Nashua Street, Suite 860, Boston, MA 02114 USA; 2 Department of Pediatrics, Harvard Medical School, Boston, MA USA; 3 Denver Health, Denver, CO USA; 4 Department of Pediatrics, University of Colorado School of Medicine, Aurora, CO USA; 5 Prisma Health, Greenville, SC USA; 6 Division of General Internal Medicine, Massachusetts General Hospital, Boston, MA USA; 7 Department of Pediatrics, Boston Medical Center, Boston, MA USA; 8 Boston University School of Medicine, Boston, MA USA; 9 Department of Pediatrics, University of South Carolina School of Medicine, Greenville, SC USA; 10 Department of Nutrition, Harvard T.H. Chan School of Public Health, Boston, MA USA

**Keywords:** Childhood obesity, Adaptations, Stakeholder engagement, Pre-implementation, Implementation science

## Abstract

**Background:**

*Connect for Health* is an evidence-based weight management program with clinical- and family-facing components for delivery in pediatric primary care for families of children ages 2 to 12 years. We used the Consolidated Framework for Implementation Research (CFIR) to guide formative work prior to national implementation. The purpose of this study was to describe the process and results of stakeholder engagement and program adaptation.

**Methods:**

We used mixed qualitative and quantitative methods to iteratively adapt and optimize the program by assessing needs and perspectives of clinicians and parents, as well as contextual barriers, facilitators, and organizational readiness for the uptake of the proposed program tools and implementation strategies. We conducted interviews with primary care clinicians from four health care organizations in Boston, MA; Denver, CO; and Greenville, SC, and used principles of immersion-crystallization for qualitative analyses. We also conducted surveys of parents of children with a body mass index ≥ 85th percentile.

**Results:**

We reached thematic saturation after 52 clinician interviews. Emergent themes representing the CFIR domains of intervention characteristics, outer and inner setting, and process included (1) importance of evidence-based clinical decision support tools that integrate into the workflow and do not extend visit time, (2) developing resources that respond to family’s needs, (3) using multimodal delivery options for family resources, (4) addressing childhood obesity while balancing competing demands, (5) emphasizing patient care rather than documentation and establishing sustainability plans, and (6) offering multiple training methods that incorporate performance feedback. Of the parents surveyed (*n* = 400), approximately 50% were Spanish-speaking and over 75% reported an annual income < $50,000. Parents affirmed the importance of addressing weight management during well-child visits, being provided with referrals and resources, and offering multiple methods for resource delivery. Decisions about program modifications were made at the program and healthcare-system level and based on stakeholder engagement findings. Modifications included cultural, geographic, and target audience adaptations, as well as varied resource delivery options.

**Conclusions:**

To ensure the fit between the *Connect for Health* program and national implementation settings, adaptations were systematically made through engagement of clinician and parent stakeholders to support adoption, sustainability, and health outcomes.

**Trial registration:**

NCT04042493

Contributions to the literature

- Few evidence-based, scalable childhood obesity interventions have been implemented in the pediatric primary care setting.
- By engaging clinician and parent stakeholders during the pre-implementation phase, we identified needs, perspectives, and preferences for the clinical- and family-facing program components, contextual barriers and facilitators, and organizational readiness, which allowed us to adapt and refine the intervention tools and implementation strategies to fit the contextual needs of the implementation sites.
- This study adds evidence about contextual factors based on stakeholder engagement that need to be evaluated prior to implementation and illustrates real-world solutions to addressing those factors.

## Introduction

Despite national obesity prevention efforts, the prevalence of childhood obesity remains high and continues to disproportionately affect low income and racial/ethnic minority children [1–3]. Primary care offers an important setting to address childhood obesity through the use of evidence-based, pediatric weight management programs (PWMP), yet few PWMPs have been translated into routine clinical practice [4–6]. Implementing childhood obesity interventions in primary care poses several challenges including time, resource, and knowledge constraints, but these barriers must be addressed to improve their adoption [4, 7]. *Connect for Health* is a proven-effective PWMP intended for delivery in primary care to improve body mass index (BMI) and family-centered outcomes that leverages clinical and community resources [8, 9]; the program is now being implemented across the USA.

The pre-implementation phase provides an opportunity to assess contextual factors that influence implementation by engaging key stakeholders, thereby increasing the likelihood of successful translation from research to practice. The Consolidated Framework for Implementation Research (CFIR) is a meta-theoretical framework that represents constructs across five domains (i.e., intervention characteristics, outer setting, inner setting, characteristics of individuals, and planning) to assist in the understanding of what works and why [10]. CFIR has been used for assessment of needs and identification of barriers and facilitators prior to implementation of programs and interventions [11]. CFIR-informed assessments in the pre-implementation phase may then result in program adaptations to suit implementation contexts, ultimately improving program effectiveness and sustainability [12, 13]. Modifications may include cultural, mode of delivery, target audience, and service setting adaptations [14], and by using a system, at this early stage, to classify type and level of modification, it can assist with future interpretation of patient, service, and implementation outcomes, and planning for sustainability and scalability [15, 16]. Formative work during the pre-implementation phase has been shown to positively influence implementation processes [17, 18], but has rarely been done for childhood obesity interventions to be delivered in primary care [19].

In preparation for national implementation of the *Connect for Health* pediatric weight management program across four healthcare organizations in the USA, we aimed to engage stakeholders, iteratively adapt and optimize the program to fit the implementation contexts, and ultimately improve the adoption of the program nationally. The purpose of this study is to describe the process and results of stakeholder engagement and program adaptation.

## Methods

### *Connect for Health* program and overview of methods

*Connect for Health* is a weight management program for families of children ages 2 to 12 years with a BMI ≥ 85th percentile that leverages clinical and community resources [8, 9]. In a randomized controlled trial, the program was shown to improve child BMI and family-centered outcomes [8]. The program includes clinical decision support tools for clinicians to facilitate screening and management and tools to support self-guided behavior change and connections to clinical and community resources for parents. Clinical-facing tools include flagging of BMI ≥ 85th percentile, decision support tools in the electronic health record (EHR) to guide management during a well child visit, and educational training materials. Family-facing tools include educational materials to support behavior change self-management, social- and community-informed text messages, and community resource guides.

**Fig. 1:**
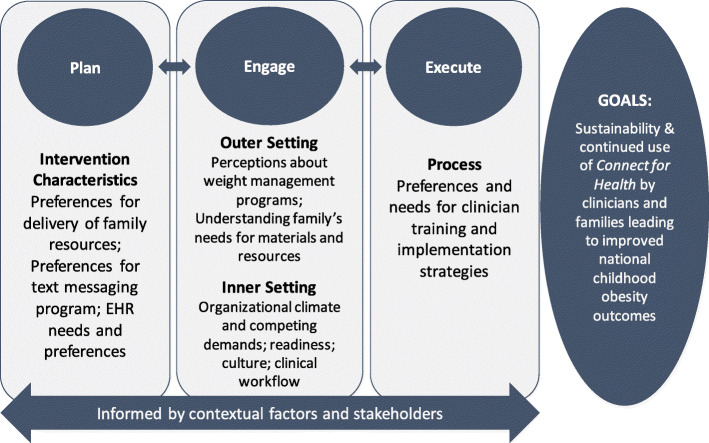
Implementation approach for *Connect for Health* drawing from the Consolidated Framework for Implementation Research

We used mixed qualitative and quantitative methods to iteratively adapt and optimize the *Connect for Health* program by assessing needs, perspectives, and preferences of clinicians and parents, as well as contextual barriers, facilitators, and organizational readiness for the uptake of the proposed program tools and implementation strategies. In preparation for implementation, we engaged pediatric clinicians and parents across four healthcare organizations through interviews and surveys consistent with the Patient Centered Outcomes Research Institute’s guidelines for stakeholder engagement [20]. In addition to clinician interviews and parent surveys, we also engaged other key stakeholders in, for example, Family and Community Advisory Council, Quality Improvement Committee, Medical Assistant Council, Information Technology Council, Community Health Board, and Clinical Unit Chief meetings. Our approach to stakeholder engagement was guided by the CFIR. Through stakeholder engagement, we explored the domains of intervention characteristics, outer setting, inner setting, and process to understand factors that would influence implementation (Fig. 1). The study protocol was approved by the Partners Health Care institutional review board and has been registered in clinicaltrials.gov (NCT04042493).

### Implementation setting and population

*Connect for Health* is being implemented in four healthcare organizations across the USA: Boston Medical Center and Massachusetts General Hospital in Boston, Massachusetts; Denver Health in Denver [21, 22], Colorado; and Prisma Health in Greenville, South Carolina. Children between the ages of 2 to 12 years with a BMI ≥ 85th percentile are eligible for the program. These sites were selected because they serve a racially-ethnically diverse, low-income population with high rates of pediatric obesity. All four healthcare organizations have pediatric practices and/or community health centers that care for children and use Epic (Verona, Wisconsin) as their EHR vendor.

### Qualitative interviews with clinicians

Pediatric clinicians and support staff were identified by research study staff and stakeholders at each site and were sent an email if they were interested in participating in an interview. Research study staff with experience conducting qualitative interviews at each site conducted interviews by phone or in-person using a semi-structured interview guide. Interviews were approximately 30–45 min in length and were audio-recorded. Participants were provided with $50 for compensation. Participants were enrolled until thematic saturation was reached and no new concepts emerged.

The interview guide was developed to understand clinicians’ needs and preferences for the clinician and family-facing program components, implementation strategies, contextual barriers and facilitators, and culture and readiness for implementation (Additional File 1). The topics aligned with the CFIR domains, including intervention characteristics, outer and inner setting, and process [10], and several questions were used from the CFIR Guide [23]. By probing these domains, we were able to identify relevant content and contextual modifications to be made to the program and implementation strategies (Fig. 1).

A professional transcription service transcribed all interviews. Using principles of immersion-crystallization [24], a team data analysis process was conducted over six iterative meetings. Immersion-crystallization is the process of reflecting on the text until insights and interpretations are reached. We completed this dynamic process by convening an analysis team that consisted of eight members with representation from all the sites and included research study staff, clinician champions, practice coaches, and information technology staff. Team members independently read the transcripts and made analytical notes. As a group, the team discussed their analyses and identified emerging themes. For each transcript, this discussion was led by varying team members. Analyses continued until no new themes emerged. The iterative discussions led to the final interpretation of the data and at the final team meeting notes from the previous meetings were compiled to assist with the interpretation. Quotes from the interviews were selected to represent themes. Group analysis notes, quotes, and final team meeting notes were compiled in Microsoft Excel. To ensure data interpretation was consistent, consensus among the team was used at all the analysis meetings.

### Parent surveys

Through an EHR search, we identified children with a BMI ≥ 85th percentile from a general pediatrics population or who attended a healthy weight clinic across our three geographic locations. Surveys were available in English and Spanish. Across all sites, surveys were completed over the phone or in-person, and data was collected and managed in the Research Electronic Data Capture (REDCap, Nashville, TN). Surveys took approximately 10 min to complete and participants received a $10 gift card as compensation.

The survey questions aligned with the CFIR domains and were intended to assist with program adaptations and implementation to ensure we met the needs of families (Additional File 2). We asked questions regarding (1) parents’ perceptions and interest in weight management programs, resources, and referrals (CFIR domain: outer setting); (2) preferences for resources and likelihood of accessing them (CFIR domain: intervention characteristics); and (3) preferences for the text messaging program and mobile phone usage and behaviors (CFIR domains: intervention characteristics and outer setting). Questions pertaining to parent’s perceptions about weight management programs were adapted from the Patient Assessment of Chronic Illness Care [25]. For each survey question, descriptive statistics were calculated, including the mean and standard deviation or frequency and percentage as appropriate. RStudio software (version 1.1.456) was used for statistical analyses [26].

## Results

### Participant characteristics

Table 1 shows the characteristics of the clinicians interviewed and parents surveyed. For the interviews, participants were predominately physicians including pediatricians, family medicine, and medicine-pediatric (84.6%), but other roles included medical assistants (7.7%), nurse practitioners (3.8%), and physician assistants (3.8%); over 75% were females. Approximately 40% of clinician interviewees were from the Boston area, 40% were from the Denver area, and approximately 20% were from the Greenville area. Of the parents who completed a survey, 37.5% were from the Boston area, 37.5% were from the Denver area, and 25% were from the Greenville area. Approximately half of parents reported their primary language spoken at home was Spanish (49.2%) and over 75% of parents reported an annual income of < $50,000.

**Table 1:** Characteristics of clinicians and parents of children ages 2–12 years with a body mass index ≥ 85th percentile

Clinician characteristics (*n* = 52)	*n* (%)
Geographic area	
Boston, Massachusetts (Boston Medical Center & Massachusetts General Hospital)	22 (42.3)
Denver, Colorado (Denver Health)	20 (38.5)
Greenville, South Carolina (Prisma Health)	10 (19.2)
Sex	
Female	40 (76.9)
Male	12 (23.1)
Clinician role	
Physician	44 (84.6)
Medical Assistant	4 (7.7)
Nurse Practitioner	2 (3.8)
Physician’s Assistant	2 (3.8)
**Parent characteristics (*n*= 400)**	***n*(%) or mean (SD)**
Geographic area	
Boston, Massachusetts (Boston Medical Center & Massachusetts General Hospital)	150 (37.5)
Denver, Colorado (Denver Health)	150 (37.5)
Greenville, South Carolina (Prisma Health)	100 (25.0)
Language spoken at home	
English	185 (46.2)
Spanish	197 (49.2)
Other	17 (4.2)
Annual income, *n* = 344	
< $20,000	84 (24.4)
$20,000 to $50,000	152 (44.2)
Greater than $50,000	63 (18.3)
Do not know	45 (13.1)
Household size, mean (SD), *n* = 397	4.28 (1.34)

### Clinician interview emergent themes

Despite differences between clinical roles, patient sociodemographics, geographic locations (i.e., urban v. rural), and workflow across the sites, we found thematic saturation after 52 clinician interviews. The findings from the engagement we completed with other key stakeholders (e.g., Quality Improvement Committee, Clinical Unit Chief meetings, Medical Assistant Council) were similar to the clinician interview findings in regards to EHR tools, workflows, and aligning with healthcare organizations’ priorities. The findings from the interviews represented the CFIR domains and resulted in six emergent themes. Table 2 shows the CFIR domains and constructs, emergent themes, and representative quotes.

**Table 2:** Emergent themes and representative quotes from clinician interviews

Themes	CFIR Constructs	Representative Quotes
Intervention characteristics		
Clinicians want evidence-based clinical decision support tools for screening and management that are actionable, integrate into their workflow, and do not detract from patient care or extend visit time.	Adaptability Complexity	“I think having the built-in processes will make it more seamless. It’s something that we can use to make sure that we’re not letting people fall through the cracks as easily, as it [might happen] if we had to remember each time ourselves.” “I think being sensitive to the potential impact of a new workflow on our existing workflow and patient experience would be nice.”
Outer setting		
Family resources should be responsive to the needs of families by being concrete, culturally sensitive, available in multiple languages, and include local resources.	Patient needs and resources	“Our community health center families…have different primary languages and primary cultures. A lot of our pediatric parents speak English, but for a lot of them, English is not their first language. The foods that [Latino families are] likely to buy are very different than what a Caucasian family or an African-American family or Cambodian family would buy. I think…the resources need to fit the health literacy of the parents.” “I personally think that it would be really nice to have something that we can hand them at the [well-child] visit, some educational materials and information about local resources… I think all of it is going to be beneficial.”
The delivery of these resources should be multimodal to suit the needs of families, clinicians, and staff.	Patient needs and resources	“I’m interested in the text messaging program. I feel like [parents] communicate that way the most. I don’t think an email would be effective… Handouts are easy for us, but not necessarily effective for the patient.”
Inner setting		
Childhood obesity is an important issue and clinicians are open to implementing new programs, but they are aware that competing priorities may detract from this program.	Implementation climate Readiness for implementation	“I think every clinic is a little bit different. Some are more open to change than others, but I think in general, we’ve done tons of new programs and have no problem. and People are generally pretty [open] as long as it’s not too much extra time.” “I wouldn't say I've never deliberately not [discussed weight management], but there's certainly times when it hasn't happened due to competing priorities or complexity of visits or a variety of things.”
For successful adoption, the program should highlight the importance of improving patient care rather than documentation, and sustainability plans should be addressed early as clinicians have seen other programs fade out.	Culture Implementation climate	“I think, for myself, and actually the other providers I work with, the practice, in general, is pretty open to new programs and changes, especially if it seems very patient-focused. I think the practice tends to drag our feet a lot on things that feel very administrative.” “I think we have a good culture of evaluation. I think people are very thoughtful about what could be better. Both, what could make the clinical practice better but also what we can do for families that is an improvement on what we're doing right now. I think that all of that is very much a part of the organization.”
Process		
Clinicians preferred a combination of in-person, individual, and online trainings that are concise, interactive, and case-based that are offered throughout the program duration and provide feedback to clinicians and practices.	Engaging Champions Reflecting and evaluating	“I think the biggest thing is going to be teaching and training. Having everybody onboard and knowing what to do… All the pieces working together would probably be the biggest thing in making sure that everybody’s onboard.” “I think the biggest ones have been… deciding what we can measure, tracking the data, and giving the feedback back to providers in a pretty timely way. When we were rolling out a project to improve our measurements for children with asthma, the clinic started off by presenting the percentage of visits where this recommended thing was happening. Then, presented what our goal target was, and gave us monthly charts that were emailed out with a lot of cheerleading for the improvements. I think it was really helpful for folks.”

*CFIR* The Consolidated Framework for Implementation Research

We asked questions about the CFIR domain of intervention characteristics regarding preferences and needs of EHR flagging and clinical decision support tools and found *clinicians wanted evidence-based clinical decision support tools for screening and management that are actionable, integrate into their workflow, and do not detract from patient care or extend visit time*. This theme provided important information regarding the CFIR constructs of intervention adaptability and complexity and allowed us to make modifications to the clinical-facing tools to be responsive to clinicians’ needs and preferences while increasing best evidence-based practice for screening and management of childhood obesity.

Two themes emerged when exploring the CFIR domain of outer setting by asking questions regarding needs and preferences for family-facing tools. The themes included (1) *family resources should be responsive to the needs of families by being concrete, culturally sensitive, available in multiple languages, and include local resources*; and (2) *the delivery of the resources should be multimodal to suit the needs of families, clinicians, and staff*. These themes highlighted patient needs and resources across the four healthcare organizations and the need to ensure the family-facing tools would be responsive to their needs.

We asked clinicians questions about implementation readiness and their organization’s culture representing the CFIR domain of inner setting and two themes emerged (1) *childhood obesity is an important issue and clinicians are open to implementing new programs, but they are aware that competing priorities may detract from this program*; and (2) *for successful adoption, the program should highlight the importance of improving patient care rather than documentation, and sustainability plans should be addressed early as clinicians have seen other programs fade out.* The first theme represented the CFIR constructs of implementation climate and organizational readiness, while the second theme represented the CFIR constructs of implementation climate and culture.

We explored the CFIR domain of process by asking questions about training and implementation strategies and we found *clinicians prefer a combination of in-person, individual, and online trainings that are concise, interactive, and case-based that are offered throughout the program duration and provide feedback to clinicians and practice*. This theme illustrated the CFIR constructs of engaging, champions, and reflecting and evaluating, and helped elucidate ways to engage clinicians in program adoption, identify key implementation leaders, and understand the role clinician champions should play in the implementation process.

### Parent survey findings

**Fig. 2:**
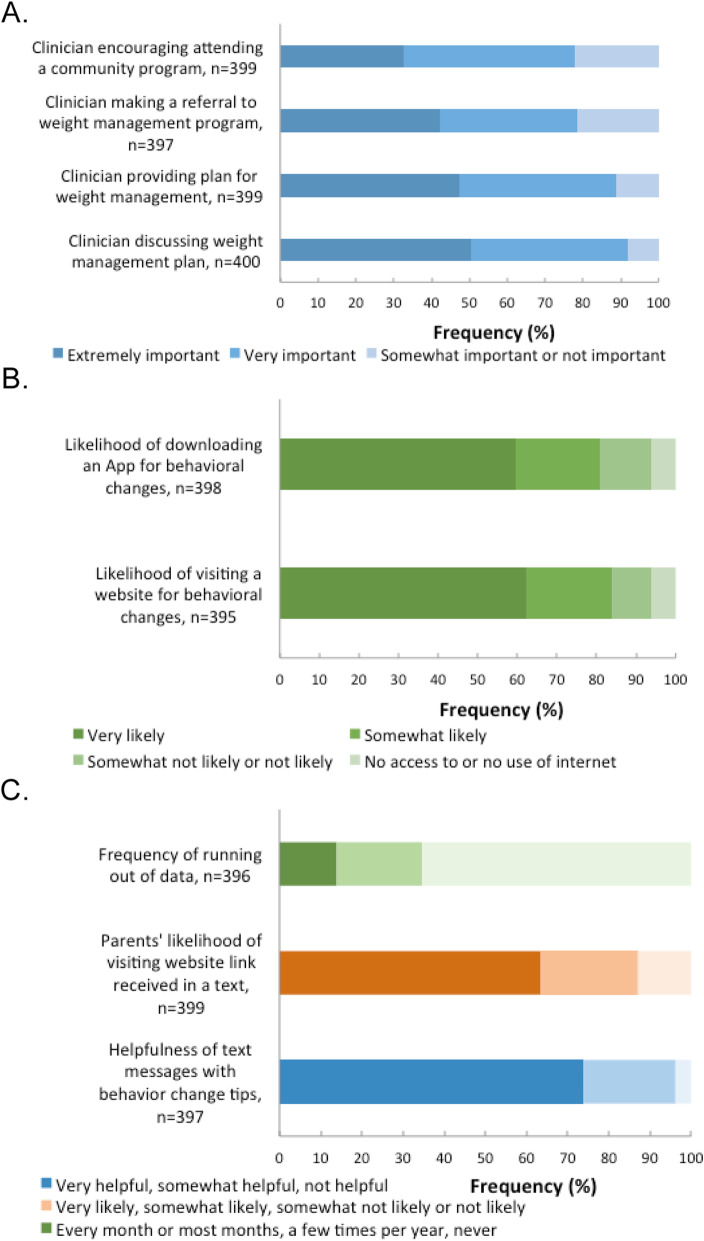
Parent perceptions of weight management programs (**a**), resource delivery methods (**b**), and text messaging preferences (**c**)

We completed 400 parent surveys (220 in English and 180 in Spanish) in the Boston, Denver, and Greenville area (see Fig. 2 and Table 3). Parents reported their perceptions of pediatric weight management programs, resources, and referrals which aligned with the CFIR construct of patient needs and resources (outer setting) and felt it was extremely or very important that their child’s primary care clinician discuss, make a plan, and provide referrals and resources related to weight management. Referrals to dieticians (30.5%) and resources about food assistance (36.8%) and structured activity programs (29.8%) were reported to be the most helpful. Parents reported their preferences for delivery of resources and likelihood of accessing resources which aligned with the CFIR construct of adaptability (intervention characteristics). We found approximately half of parents preferred after-visit summaries to be printed while at their child’s visit and approximately half of families wanted other resources, such as educational materials and other content, to be texted to them. Parents reported being willing to download an app (59.5%) or visit websites (62.3%) to find additional information about behavioral changes. The questions about preferences for the text messaging program and mobile phone usage and behaviors aligned with the CFIR constructs of adaptability (intervention characteristics) and patient needs and resources (outer setting). We found nearly three-quarters of families thought it would be helpful to receive text messages with behavioral change tips and reported being willing to follow links to access additional content. Approximately 30% of families reported running out of data on their mobile phone. The findings from engagement with parent stakeholders through family and community advisory councils were similar to the findings of the surveys. For example, parents expressed interest in the educational topics covered in the program and preferred multi-modal delivery of materials and resources. Findings across sites were similar and differences did not drive any modifications.

**Table 3:** Parent survey results: perceptions of referrals, resources, and delivery methods

	*n* (%)
**Referrals and resources for weight management**	
**Helpful referrals that a clinician has made**^a^	
Nutritionist/dietician	122 (30.5)
Weight management program or clinic	76 (19.0)
Other (i.e., Cooking classes, websites, apps)	75 (18.7)
YMCA or Boys & Girls Club	52 (13.0)
Specialist (i.e., gastroenterologist, endocrinologist)	51 (12.8)
None of the above	193 (48.2)
**Resources parents have found to be helpful**^**b**^	
Women, Infants, and Children (WIC) or Supplemental Nutritional Assistance program (SNAP)	147 (36.8)
Structured activity programs (i.e., dance, soccer)	119 (29.8)
Primary care provider	89 (22.2)
Nutritionist/dietician	74 (18.5)
School programs	63 (15.8)
YMCA or Boys & Girls Club	54 (13.5)
Weight management program or clinic	47 (11.8)
Other (i.e., websites, apps)	42 (10.6)
Farmer’s market or food bank	36 (9.8)
None of the above	66 (16.5)
**Parents’ preferences for resource delivery**	
**Delivery preference for an after-visit summary,*n*= 399**	
Printed at doctor’s office	235 (58.9)
Mailed home	51 (12.8)
Emailed	51 (12.8)
Sent using the Patient Portal (i.e., MyChart)	40 (10.0)
From a text that has a link to the after visit summary	20 (5.0)
Other	2 (0.5)
**Preferred way to receive educational handouts**^**b**^	
Printed at doctor’s office	232 (58.0)
SMS text with link to handout	190 (47.5)
Emailed	167 (41.8)
Patient Portal	75 (18.8)
Text messaging app (i.e., WhatsApp)	64 (16.0)
Other (i.e., social media, website, app)	68 (16.9)
**Preference for delivery of behavior change content,*n*= 399**	
Text messages	205 (51.4)
Email	123 (30.8)
Other (i.e., WhatsApp, social media)	71 (17.8)

^a^Parents could choose more than one response

^b^Parents could choose up to three responses

### *Connect for Health* program adaptations

Following the mixed methods assessments, we made several adaptations to the core program components and to the implementation strategies. We found the results of the engagement across all stakeholders converged which strengthened our modification decisions. As a research team across all four organizations, we collectively reviewed results of the clinician interviews, parent surveys, and other stakeholder engagement activities. Based on the findings, we made modifications at the program level and then each healthcare organization identified additional changes for their setting. During the pre-implementation phase, the research team met monthly via video conferencing, as well as an in-person meeting to review and discuss program adaptations. Across the core components, we identified cultural and geographic, mode of delivery, and target audience adaptations that were necessary due to differences in geographic location and patient populations as compared to the original trial, differences in organizational culture and clinical workflow, and in consideration of future program scalability [14]. Table 4 shows the nature of the content modifications, level of delivery of the modifications, and by whom made the modifications using Stirman and colleagues’ adaptation classification system [15, 16]. This adaptation classification system was used to assist with future interpretation of patient, service, and implementation outcomes, and sustainability and scalability planning.

**Table 4:** Classifying adaptations to the *Connect for Health* pediatric weight management program

Core components	What are the modifications?	At what delivery level was the modification made?	Who made the decision to modify?
Flagging of children with elevated BMIs	Changed from interruptive BPA to non-interruptive BPA	Healthcare system level customized for each organization	Based on stakeholder engagement
	Changed to who received the BPA depending on workflow of healthcare system (i.e., physician v. medical assistant)	Healthcare system level customized for each organization	Based on stakeholder engagement
	Additional content and actions included in the BPA depending on healthcare system’s needs	Healthcare system level customized for each organization	Based on stakeholder engagement
Clinical decision support tools	Patient education materials, community resource guide, and clinician educational materials accessible through EHR	Healthcare system level customized for each organization	Based on stakeholder engagement
	Enrollment for text messaging program through order as part of clinical decision support tools. In the original trial, parents were enrolled by a health coach	Program level across all sites	Program developer
	Aligned the clinical decision support tools with internal performance metrics	Healthcare system level customized for each organization	Based on stakeholder engagement
Patient education materials	Materials translated into Spanish and Haitian Creole. In the original trial, materials were only available in English	Program level across all sites	Program developer
	Consolidated patient educational materials into one page handouts per behavior	Program level across all sites	Program developer
	Revised to be geographically and culturally appropriate	Program level across all sites	Based on stakeholder engagement
	Addition of “Establish a balanced nutrition plan” as a primary behavioral goal with a corresponding handout	Program level across all sites	Program developer
Community resource guide	Customized for each healthcare system and for health centers within each system	Healthcare system level customized for each organization	Program developer
	Created an additional one page handout of top resources for each practice	Healthcare system level customized for each organization	Based on stakeholder engagement
Text messaging	Messages revised to be geographically and culturally appropriate	Program level across all sites	Based on stakeholder engagement
	Messages revised to be unidirectional v. bidirectional	Program level across all sites	Program developer
Health coach	Health coaching component of program removed. Information incorporated into educational materials, community resource guide, and text messaging program	Program level across all sites	Program developer
Implementation strategies	Selected clinician champions who are embedded within the clinical practices to facilitate implementation by engaging other clinicians and providing support and feedback	Program level across all sites	Program developer/ Based on stakeholder engagement
	Added practice coaches to provide clinicians with “at the elbow” support	Program level across all sites	Program developer
	In-person trainings to include all practice staff (i.e., clinicians, medical assistants, and front-desk staff) and to occur throughout the implementation period for continued education and feedback	Program level across all sites	Based on stakeholder engagement
	Offered continuing educational units and quality improvement bonuses to incentive trainings and likelihood that all clinicians would be familiar with the program	Healthcare system level customized for each organization	Based on stakeholder engagement
	Added a virtual learning community to provide on-demand support to clinicians with best practice management of childhood obesity	Program level across all sites	Based on stakeholder engagement

*BMI* body mass index, *BPA* best practice alert, *EHR* electronic health record

Based on the interviews, we learned each of the healthcare organizations had their own unique practice workflow and had customized EHR instances; therefore, each site modified the flagging of children with an elevated BMI to fit their needs. For example, changes included creating a non-interruptive best practice alert and having medical assistants receive alerts rather than the physician. Changes to the clinical decision support tools included housing family-facing tools within the EHR and creating an order to enroll patients in the text messaging program that would support future program sustainability. We aligned changes to the best practice alert and clinical decision support tools with performance metrics at the healthcare organizations to incentivize the usage of the EHR tools and for sustainability purposes.

Modifications to the family-facing tools (i.e., patient educational materials, community resource guide, and text messaging program) included translation of materials into languages spoken by the healthcare organizations’ patient populations, revision to the materials to meet the needs of patients in urban and rural settings and geographic locations across the USA, and consolidation of educational materials for ease of delivery. Additional changes included a handout focused on creating a balanced nutrition plan and modifying the text messages to be unidirectional rather than bidirectional. Several of these modifications (i.e., consolidation of materials, unidirectional text messages, elimination of health coach) were made at the program-level in consideration of program sustainability and scalability.

Modifications to the implementation strategies included selecting a clinician who was embedded within the clinical practices to engage other clinicians and champion the program, adding a practice coach to provide technical assistance, and inviting all staff to in-person trainings and extending them throughout the implementation phase. Based on the interest in on-demand trainings, we also added a virtual learning community for capacity building of childhood obesity screening and management. For the in-person trainings and virtual learning community, we intend to offer continuing educational units and quality improvement bonuses (in collaboration with other hospital departments as available) to incentive participation and increase the likelihood that clinicians will be familiar with the program. Modifications to the implementation strategies were made at the program level, but each of the healthcare organizations were encouraged to customize as needed.

## Discussion

In this mixed methods study involving stakeholder engagement to adapt the *Connect for Health* pediatric weight management program, six themes emerged that informed adaptations to the clinical- and family-facing components of the program that mapped to the CFIR domains of intervention characteristics, outer setting, inner setting, and process. Additionally, parents affirmed the importance of addressing weight management during well-child visits, being provided with referrals and resources, and delivering resources using a variety of methods. We leveraged the stakeholder feedback to then iteratively modify the program in preparation for national implementation. A key component of program implementation has been maintaining the core components of the evidence-based intervention while adapting it to each site’s workflow, needs, and culture. This approach makes the program generalizable to a broad variety of medical homes that serve children, including hospital-based clinic systems, federally qualified health centers, and other clinic-based systems. The approach also serves as a model for disseminating and implementing other clinical practice innovations.

The high prevalence of childhood obesity necessitates the translation of evidence-based interventions into routine clinical care to improve child health outcomes. Effective pediatric weight management interventions are being implemented in school-systems, community programs, and child-care programs [27–30], but few have been implemented into the primary care setting [19]. The primary care setting offers many opportunities, as most children annually attend a well-child visit and families look to clinicians for health advice and support, but this setting also has many challenges [4, 7]. We found that clinicians do want evidence-based programs to effectively manage childhood obesity, but the program must be responsive to their needs. To do this, we engaged stakeholders which have been shown to be an effective method for understanding practice needs and informing adaptations [31]. Studies have used stakeholders to adapt and refine program materials, develop implementation strategies, and plan for sustainability resulting in improved implementation and health outcomes [30, 32–34].

Using the CFIR, we explored characteristics of the *Connect for Health* program, the outer setting, the inner setting at the four healthcare organizations, and the implementation process. Clinicians discussed that complexity and adaptability of the intervention would impact their adoption of this program. For example, if the EHR tools did not integrate into their existing workflow or if the tools added additional time or “clicks” to the visit, clinicians reported they would be reluctant to use the program. These contextual factors have been previously identified, but through our engagement, we could probe for solutions and make adaptations to the EHR tools and implementation strategies [35]. Several adaptations we made aligned with Ross and colleagues’ recommendations for implementation from their systematic review of contextual factors that affect the uptake of e-health technologies [36]. These included training prior to “go live” and throughout the implementation process, engaging key stakeholders, ensuring buy-in from administration, and appointing clinician champions.

Engaging parents helped us to understand their needs and refine the program and our implementation plans. Findings from the clinician interviews and parent surveys aligned, including multimodal delivery methods of materials, parent’s interest in discussing and developing plans for weight management, and providing referrals and resources. In addition, clinicians discussed the importance of family-facing resources and materials being responsive to family’s needs, culture, and geographic location. This information and the resultant modifications ensured that our materials were family-centered. Interventions that are family-centered have been shown to improve child health outcomes [37], but many pediatric weight management interventions are not family-centered [38], reinforcing the importance of matching the program components with the needs of patients to increase implementation success.

Understanding culture, implementation climate, and organizational readiness assisted with planning and developing implementation strategies. Clinicians discussed methods of how they wanted to be engaged in adopting this program. Methods included in-person and on-demand educational trainings that were offered both individually and as a group. Clinicians also recognized the importance of receiving feedback throughout the implementation phase to improve their performance. To meet this need, we will have clinician champions deliver feedback and encourage reflection and evaluation. A systematic review found that champions were associated with implementation success, although the findings suggest that it was champions in conjunction with other strategies that promoted change [39]. For implementation of the *Connect for Health* program, clinician champions will encourage reflection and evaluation, but other strategies will be employed, including practice coaching and learning communities to promote adoption through other mechanisms of action [40]. We have identified a core group of implementation strategies, but given the unique inner setting of each of the healthcare organizations, we are encouraging sites to tailor strategies as needed which will be assessed and modified throughout the implementation process.

This formative work during the pre-implementation phase represents an important step in the implementation of *Connect for Health* in four healthcare organizations; this work, though, does present with limitations. In the clinician interviews, we did not include questions pertaining to the CFIR domain of characteristics of individuals. Although this domain may offer insights into the uptake of the program, we felt that during this phase, other domains were more critical for planning and we choose to omit these questions in the interview guide. Additionally, we were not able to interview all of the unit chiefs of the health centers or practices. To ensure buy-in and that we understood the needs and culture of the individual health centers and practices, we attended meetings with unit chiefs, both individually and as a group. The four healthcare organizations whom will be implementing *Connect for Health* have several differences (i.e., geographic locations, patient populations), but also share commonalities including being large university-affiliated medical centers and having the same EHR vendor. Therefore, some of the findings from the parent surveys and clinician interviews may not pertain to other healthcare settings.

## Conclusion

Given the high prevalence of childhood obesity and its disproportionate impact on racial and ethnic minority, low-income populations, the adoption of the evidence-based *Connect for Health* program in the pediatric primary care setting stands to improve the health and family-centered outcomes of children throughout the USA. In preparation for national implementation of the *Connect for Health* pediatric weight management program, we engaged clinician and parent stakeholders through a mixed methods assessment. This approach during the pre-implementation phase was useful in adapting and refining the program and implementation strategies to fit the contextual needs of the four healthcare organizations. Through engagement, we identified needs and preferences for the clinical- and family-facing program components, contextual barriers and facilitators, and organizational readiness, which resulted in cultural, mode of delivery, and target audience adaptations at the program and healthcare system level. During the implementation phase, we will evaluate the effectiveness of our implementation strategies to increase the uptake of this program in the pediatric primary care setting.

## Supplementary information

**Additional File 1: ***Connect for Health* Clinician Interview Guide. Description of data: This supplemental file contains questions used to guide interviews with clinicians prior to program implementation across sites.

**Additional File 2: ***Connect for Health* Parent Feedback Survey. Description of data: This additional file contains survey questions used across implementation sites to solicit feedback from parents regarding the *Connect for Health* pediatric weight management program.

## Data Availability

The datasets used during the current study are available from the corresponding author on reasonable request.

## Abbreviations

PWMP: Pediatric weight management programs
BMI: Body mass index
CFIR: Consolidate Framework for Implementation Research
EHR: Electronic health record
