# Supplementary material for: Engaging stakeholders in the adaptation of the Connect for Health pediatric weight management program for national implementation
Source: Implement Sci Commun. 2020 Jun 17;1:55. doi: 10.1186/s43058-020-00047-z (PMC7427919; doi:10.1186/s43058-020-00047-z)
Supplement: Supplementary file 1 — Additional File 1: Connect for Health Clinician Interview Guide. Description of data: This supplemental file contains questions used to guide interviews with clinicians prior to program implementation across sites. [file 43058_2020_47_MOESM1_ESM.docx]

*Connect for Health* is a pediatric weight management program for children ages 2-12 who have a BMI ≥85^th^ percentile. The program is based on a randomized trial that showed improvements in children’s body mass index. The goal of this interview is to learn how to increase the adoption of the program in your primary care practice.

**Introductory Questions**

I’d like to start by learning more about your experience working with children with obesity.

1. In general, what tools in the clinical setting help with your management of children with obesity? Are there any plug-ins or features in Epic that have made taking care of patients with obesity more seamless? Do you have examples of other tools you use beyond obesity that you have found helpful for management of children with specific health conditions?

**Overall Program Feedback**

1. Now I would like to hear your thoughts on the program components. The *Connect for Health* program includes electronic health record enhancements, including clinical decision support tools for the flagging, management and follow-up of children with obesity. It also includes training for clinicians and medical staff, parent educational materials, and family text messages to support behavior change. As you think about the components just listed:
   1. What are your initial thoughts about the program components?
   2. Are there any additional components you think would be helpful to include?

**EHR Alert**

Now I’d like to ask a few questions about proposed enhancements to the electronic health record for screening, management, and follow-up of children with obesity.

1. Best practice for childhood obesity management includes flagging children who have a BMI ≥ [85^th^/95^th^] percentile, notifying clinicians within the health record, and providing supportive tools to guide screening and management. In the original *Connect for Health* trial we used a BPA (Best Practice Alert) within Epic. We would also like to explore other ideas.
   1. What do you think would be the best way to flag a child as having an elevated BMI?

Probe: When is an optimal time to flag or alert you to a child with an elevated BMI?

- 1. What barriers do you see in using a BPA?
     1. What useful components could be put into the system to make the BPA worth using instead of ignoring it?

**EHR Program Components**

1. The *Connect for Health* program will include information about labs, referrals, follow-up, an after-visit summary and patient education materials. I’d like to hear your thoughts on each, specifically what you find helpful in managing children with obesity.
   1. Labs, Referrals, Follow-up
      1. Are there labs, referrals, follow-up that you would want to make sure we include?
   2. After-Visit Summary & Patient Education Materials
      1. The program includes an after-visit summary. Do you currently use after-visit summaries? What format do you use to give this to your patients? Is there a format you would prefer?

Probes: Printed out at the visit, Sent via MyChart/Patient Gateway

- - 1. The educational materials will cover topics such as healthy eating habits, screen time, physical activity, sleep, and stress. Are there topics you would like to see added or removed?
    2. What would be the easiest way for you to provide these materials to families? For example, print the materials, send to MyChart/ Patient Gateway, text or email the materials, direct link to materials at a website, create an App for families.

**Text Messaging**

1. *Connect for Health* includes text messaging for families. Parents enrolled in the program receive text messages that focus on supporting family behavior change over the course of one year.
   1. Do you think your patients will be interested in enrolling?
2. What barriers do you think might prevent patients from signing up for the text messaging program?
3. What would be the best way to enroll families to receive the text messages?
   1. What would be the best way to confirm the mobile phone number?

**Clinician Training**

1. Now we would like to talk with you about our plan for helping clinicians adopt the program. Your practice will have on-site support from a clinician champion and a practice coach to demonstrate how to use the tools. We will also provide an overview of the tools and how to manage children with obesity at regularly scheduled staff meetings and through a virtual, on-line learning community.

- 1. Are there particular strategies that you would find helpful in learning about the *Connect for Heath* Program or learning about best practices for screening and management of children with obesity?
     1. Would you participate in a virtual, on-line learning community? If yes, how often? What information would you want covered?
     2. How could a clinician champion or practice coach best support you?
  2. Are there other resources, topics or components you would like to add to the training plan?
     1. Probes: Information about healthy lifestyles, Manuals and webinars for using the EHR enhancements, access to webinars about quality improvement

**Readiness for Change/ Implementation Climate**

I’d like to now ask a few questions about your organization’s readiness to adopt a program like *Connect for Health*.

1. To what extent are new ideas and programs embraced and used to make improvements in your organization?
2. To what extent might this program take a backseat to other high-priority initiatives going on now?

**Wrap-Up**

I’d like to end with a few wrap-up questions.

1. Do you see any possible barriers to adopting this program? How could we address those barriers?
2. Anything else you would like to share with us?
