# Supplementary material for: Engaging stakeholders in the adaptation of the Connect for Health pediatric weight management program for national implementation
Source: Implement Sci Commun. 2020 Jun 17;1:55. doi: 10.1186/s43058-020-00047-z (PMC7427919; doi:10.1186/s43058-020-00047-z)
Supplement: Supplementary file 2 — Additional File 2: Connect for Health Parent Feedback Survey. Description of data: This additional file contains survey questions used across implementation sites to solicit feedback from parents regarding the Connect for Health pediatric weight management program. [file 43058_2020_47_MOESM2_ESM.docx]

*Connect for Health is a pediatric weight management program that helps children achieve a healthy weight. We will describe to you parts of this program and we would like to know what you think.*

1. *As part of this program, your provider will talk with you about how you and your child can make changes to achieve a healthy weight.*

- How important is it to you that your child’s provider talks about a plan to help your child get to a healthy weight?

❑ Extremely Important

❑ Very Important

❑ Somewhat Important

❑ Not Important

1. *Your provider might make referrals to specialists or to community programs. We would like to know what programs and places have helped your family in the past and what programs and places might be helpful to you.*

- What programs and places has your provider referred you to that have helped your child achieve a healthy weight? (Pick all that apply)

❑ Nutritionist/Dietician

❑ Weight Management Program or Clinic

❑ Specialist such as a gastroenterologist or endocrinologist

❑ Websites or Apps

❑ YMCA or Boys & Girls Club

❑ Cooking classes

❑Other: __________________________________

❑ None of the above

- What programs and places have you found to be helpful in supporting your child’s weight management? Pick three most important resources.

❑ SNAP (food stamps)

❑ WIC

❑ Nutritionist/Dietician

❑ Primary care provider

❑ Weight Management Program or Clinic

❑Websites or Apps

❑ Food bank

❑ Farmer’s Market

❑ Structured activity programs such as dance class or soccer team

❑ School programs

❑ YMCA or Boys & Girls Club

❑ Other: __________________________________

❑ None of the above

- How important is it to you that your child’s provider sends you to a specific clinic or program to help your child get to a healthy weight?

❑ Extremely Important

❑ Very Important

❑ Somewhat Important

❑ Not Important

- How important is it to you that your child’s provider encourages you to attend programs in the community (such as the YMCA or Boys and Girls Club) to help your child get to a healthy weight?

❑ Extremely Important

❑ Very Important

❑ Somewhat Important

❑ Not Important

1. *After your child’s provider visit, they will give you a written summary (also known as an after-visit summary) of what was talked about during the visit and any recommendations they have for you. We would like to know more about the after-visit summary.*

- How important is it to you that your child’s provider gives you a specific plan to help your child get to a healthy weight?

❑ Extremely Important

❑ Very Important

❑ Somewhat Important

❑ Not Important

- How would you want to get the after-visit summary?

❑ Printed in the doctor’s office

❑ Mailed to me

❑ Emailed to me

❑ From a text that has a link to the after-visit summary

❑ Sent using the Patient Portal (MyChart, Patient Gateway)

❑ Other: __________________________________

- Do you use the Patient Portal (MyChart, Patient Gateway)?

❑ Yes

❑ No

❑ Don’t know

1. *The Connect for Health program provides educational materials and text messages to families. We would like to know more about how you would like to get this kind of information and how you would use it.*

- How would you want to receive educational handouts? Pick three most preferred ways.

❑ Text messages with a link to the handouts

❑ Text messaging apps (Whats App)

❑ Email

❑ Patient Portal (MyChart, Patient Gateway)

❑ Website

❑ App

❑ Social media (Twitter, Instagram, Facebook, Snap Chat)

❑ Printed handouts in the doctor’s office

- - If printed handouts are selected: If the handouts had a link to a website, would you visit that website?

❑ Yes

❑ No

- How likely are you to visit a website that has information about making changes to help your child achieve a healthy weight?

❑ Very likely

❑ Somewhat likely

❑ Somewhat not likely

❑ Not likely

❑ I don’t have access to or don’t use the internet

- How likely are you to download and use an App that has information about making changes to help your child achieve a healthy weight?

❑ Very likely

❑ Somewhat likely

❑ Somewhat not likely

❑ Not likely

❑ I don’t have access to or don’t use the internet

- How likely are you to open a link to a webpage or video with more information if it was included in a text message?

❑ Very likely

❑ Somewhat likely

❑ Somewhat not likely

❑ Not likely

- How would you want to get messages with tips about making behavior changes?

❑ Text messages

❑ Text messaging app (What’s App)

❑ Direct messaging (Facebook, Instagram, Snap Chat)

❑ Email

❑ Other: ___________________________

- How often do you run out of data on your phone?

❑ Always (every month)

❑ Often (most months)

❑ Sometimes (a few times per year)

❑Never

- What time of day would you be most likely to read a text?

❑ Morning

❑ Afternoon

❑ Evening

- How often would you want to get text messages that have tips about making behavior changes?

❑ 1 time a week

❑ 2 times a week

❑ More than 2 times a week

- How helpful do you think text messages with tips about making behavior changes would be?

❑ Very helpful

❑ Somewhat helpful

❑ Not helpful

1. *We would like to know more about your household. Your answers are private and will not be shared with anyone outside of the research team.*

- What is your preferred language at home?

❑ English

❑ Spanish

❑ Other: __________

- How many people live in your household, including yourself?

__ __ people

- What is your annual household income from all sources?

❑ Less than $10,000 per year

❑ $10,000 to $15,000 per year

❑ $15,001 to $20,000 per year

❑ $20,001 to $25,000 per year

❑ $25,001 to $35,000 per year

❑ $35,001 to $50,000 per year

❑ $50,001 to $75,000 per year

❑ Greater than $75,000 per year

❑ Don’t Know
